# Metabolomic Profiling Identifies Exogenous and Microbiota-Derived Metabolites as Markers of Methotrexate Efficacy in Juvenile Idiopathic Arthritis

**DOI:** 10.3389/fphar.2021.768599

**Published:** 2021-12-09

**Authors:** Ryan Sol Funk, Mara L. Becker

**Affiliations:** ^1^ Department of Pharmacy Practice, University of Kansas Medical Center, Kansas City, KS, United States; ^2^ Department of Pediatrics, Division of Rheumatology, Duke University School of Medicine, Durham, NC, United States

**Keywords:** juvenile idiopathic arthritis, methotrexate, metabolomic, biomarker, pediatric, pharmacology

## Abstract

Variability in methotrexate (MTX) efficacy represents a barrier to early and effective disease control in the treatment of juvenile idiopathic arthritis (JIA). This work seeks to understand the impact of MTX on the plasma metabolome and to identify metabolic biomarkers of MTX efficacy in a prospective cohort of children with JIA. Plasma samples from a cohort of children with JIA (*n* = 30) collected prior to the initiation of MTX and after 3 months of therapy were analyzed using a semi-targeted global metabolomic platform detecting 673 metabolites across a diversity of biochemical classes. Disease activity was measured using the 71-joint count juvenile arthritis disease activity score (JADAS-71) and clinical response to MTX was based on achievement of ACR Pedi 70 response. Metabolomic analysis identified 50 metabolites from diverse biochemical classes that were altered following the initiation of MTX (*p* < 0.05) with 15 metabolites reaching a false-discovery rate adjusted p-value (q-value) of less than 0.05. Enrichment analysis identified a class-wide reduction in unsaturated triglycerides following initiation of MTX (q = 0.0009). Twelve of the identified metabolites were significantly associated with disease activity by JADAS-71. Reductions in three metabolites were found to be associated with clinical response by ACR Pedi 70 response criteria and represented several microbiota and exogenously derived metabolites including: dehydrocholic acid, biotin, and 4-picoline. These findings support diverse metabolic changes following initiation of MTX in children with JIA and identify metabolites associated with microbial metabolism and exogenous sources associated with MTX efficacy.

## Introduction

Rapid control of disease activity to prevent irreversible joint damage and improve long-term outcomes continues to be a major therapeutic goal in the treatment of juvenile idiopathic arthritis (JIA) (Consolaro et al., 2012). Response to drug therapy is currently characterized by a variable and unpredictable course, which commonly necessitates a trial-and-error treatment approach to identify an effective treatment regimen (Becker, 2012). In the management of JIA, methotrexate (MTX) has continued to be a cornerstone of therapy since the establishment of its efficacy in the early 1990s (Giannini et al., 1992). However, MTX is characterized by a delayed onset of action with approximately one in three patients failing to adequately respond to initial treatment (Ruperto et al., 2004). At this time, no reliable biomarkers exist to guide drug selection and optimization and the current approach wastes precious time and jeopardizes the overarching goal of early disease control (Funk and Becker, 2016). As a result, there exists a need to apply novel analytical approaches to further understand the pharmacology of MTX and to identify biomarkers to guide clinicians in the selection and optimization of drug therapy.

Efforts to identify clinical biomarkers of MTX response in JIA have predominately focused on targeted analyses related to the disposition of MTX, its intracellular metabolism to form polyglutamated metabolites, and its pharmacological effects on folate and folate-dependent biochemical pathways (Funk et al., 2014; Calasan et al., 2015). A major limitation to this targeted approach has been the biased nature of these studies, which often limit that analysis to only a handful of metabolites representing a limited number of biochemical pathways. As an -omics approach, metabolomics offers a relatively unbiased method to investigate the effect of MTX on a diversity of biochemicals and biochemical pathways towards an improved understanding of the pharmacological basis for response to MTX and to identify potential metabolic biomarkers of MTX response in JIA (Funk et al., 2020).

The metabolome is the complete set of low-molecular weight chemicals within a biological system and represents not only the activities of genetically encoded metabolic pathways, but also reflects exogenous exposures (e.g., diet, drugs, vitamins, toxins, microbiota) (Fiehn, 2002). Metabolomics is the study of these molecules within that system. In the case of patients, the plasma or serum metabolome is commonly measured because of the relative ease of collection, preparation, and storage (Beger et al., 2016). Metabolomic studies have been conducted in patients with rheumatoid arthritis (RA) and other autoimmune diseases and have identified several metabolites and metabolic pathways associated with disease activity and drug response (Wang et al., 2012; Jiang et al., 2013; Kapoor et al., 2013; Young et al., 2013; Guma et al., 2016). However, this work represents the first to evaluate metabolomic changes resulting from the initiation of MTX in JIA and the association of these changes with the drug’s early clinical efficacy.

In this exploratory study, a semi-targeted global metabolomics approach is applied to evaluate metabolomic changes associated with the initiation of MTX in a prospective cohort of children with JIA. The resulting metabolomic data is subsequently used to identify potential metabolic biomarkers of MTX response in these patients and novel biochemical pathways relevant to MTX efficacy in JIA. The metabolomic profiles are evaluated using chemometric and network enrichment analyses to interpret the impact of MTX on the plasma metabolome and they are then further integrated with the clinical response data to identify putative metabolomic markers of MTX response in JIA.

## Materials and Methods

### Patients

Plasma samples were acquired from a single-center prospective cohort of JIA patients. The study included patients diagnosed with JIA requiring initiation of MTX monotherapy as deemed appropriate by their treating provider. Patients who required a concurrent biologic disease modifying antirheumatic drug (DMARD) were excluded. Diagnosis of JIA was established based on the Edmonton 2001 International League of Associations of Rheumatology (ILAR) criteria for JIA (Petty et al., 2004). Patients were initiated on a standardized MTX dose of 15 mg/m^2^ weekly along with 1 mg folic acid daily. MTX route of administration for the study was standardized to subcutaneous, however due to a national shortage of MTX for injection, oral MTX was permitted (*n* = 12). All patients completed 3 months of full and consistent dosing of 15 mg/m^2^ of MTX weekly prior to their follow up sample collection. Concurrent medications allowed included nonsteroidal anti-inflammatory drugs (NSAIDs) and daily low-dose corticosteroids (the lesser of 0.2 mg/kg/day or 10 mg of prednisone). Intra-articular corticosteroid injections (IASI) were allowed, however injected joints were counted as “active” at follow up to reduce the potential bias resulting from IASI. Venous blood samples (10 ml) were collected in K2-EDTA containing tubes prior to initiation of MTX and at the patient’s routine 3-months follow up clinic visit along with routine laboratory blood draws. Parental/patient (>18 years of age) written informed consent and patient written informed assent for patients ages 7–18 years were collected from all participants in accordance with approval from the Children’s Mercy Hospital Pediatric Institutional Review Board.

### Clinical Data

Clinical response data collected at 3 months included the American College of Rheumatology (ACR) Pediatric 30, 50, and 70 response, which is a composite score comprised of: 1) physician global assessment of disease activity (MD-VAS), 2) patient/parent assessment of overall well-being (PT-VAS), 3) functional ability (Childhood Health Assessment Questionnaire, CHAQ), 4) number of joints with active arthritis, 5) number of joints with limited range of motion, and 6) erythrocyte sedimentation rate (ESR) (Giannini et al., 1997). We also utilized the validated continuous Juvenile Arthritis disease Activity Score (JADAS-71) compiled from the normalized ESR, the active joint count, PT-VAS, and MD-VAS (Consolaro et al., 2009). Additional variables collected included age, sex, serum c-reactive protein (CRP) levels, MTX route of administration, NSAID use, and oral corticosteroid use.

### Metabolomics Analysis

Plasma was isolated from venous blood samples by centrifugation at 2,000 RPM in a Beckman tabletop centrifuge for 10 min. The resulting plasma supernatant was separated into aliquots and stored at -80°C prior to analysis. Samples were shipped on dry ice to the NIH West Coast Metabolomics Center at the University of California, Davis (Davis, CA) for global semi-targeted analysis utilizing three independent standardized analytical methods to measure intermediates of primary metabolism, biogenic amines, and lipids (Cajka and Fiehn, 2016; Fiehn, 2016). Samples were thawed and prepared for analysis using a biphasic liquid-liquid extraction method developed for metabolomic analysis using plasma and serum samples across the three analytical platforms, which includes automated liner exchange-cold injection system gas chromatography time-of-flight mass spectrometry, charged surface hybrid chromatography electrospray ionization quadropole time-of-flight mass spectrometry, and hydrophilic interaction liquid chromatography electrospray ionization quadropole time-of-flight mass spectrometry. The resulting peaks were identified based on retention times and mass spectra from MassBank of North America and reported as peak height intensities. Peak height intensity tables were curated by the NIH West Coast Metabolomics Center and submitted to Metabolomics Workbench (https://www.metabolomicsworkbench.org/) under Project ID PR001148. Raw peak intensity data from each analytical platform underwent a standardized normalization procedure. The normalization ratio was calculated as the ratio of the sum of peak heights for all identified metabolites (mTIC) for each sample to the total average mTIC for all samples. Peak heights for each metabolite were divided by the normalization ratio to arrive at the normalized peak height intensity. Metabolites measured in more than one analytical platform were combined by mean normalization to give equal weighting to each platform and averaged. The resulting normalized peak height intensities for identified compounds were uploaded into MetaboAnalyst 3.0 and normalized by logarithmic transformation and Pareto scaling (Xia et al., 2015; Xia and Wishart, 2016). The resulting data were analyzed for fold-change and non-parametric paired analysis and visualized using volcano plots to identify metabolites significantly altered following the initiation of MTX.

### Enrichment Analysis

The fold-change and p-values for each identified metabolite were extracted and used to conduct chemical and metabolic network enrichment analysis. Visualization of chemometric and biochemical networks altered following the initiation of MTX was conducted using MetaMapp to generate a network map based on chemical similarity utilizing the Kyoto Encyclopedia of Genes and Genomes (KEGG) metabolic network database and Tanimoto substructure similarity coefficients (Barupal et al., 2012). The resulting mapping data was uploaded into Cytoscape 3.7.1 for visualization. Enrichment analysis based on chemical similarity was also conducted using the open-source software Chemical Similarity Enrichment Analysis for Metabolomics (ChemRICH) (Barupal and Fiehn, 2017). The ChemRICH analysis uses Tanimoto substructure similarity coefficients and medical subject headings ontology to generate non-overlapping clusters of metabolites into distinct chemical classes and is independent of biochemical pathway assignments.

### Statistical Analysis

Analysis of changes in individual metabolites following the initiation of MTX was conducted in MetaboAnalyst 3.0 using paired non-parametric univariate analysis. All metabolites achieving a raw p-value less than 0.05 were used in the chemometric and metabolic network enrichment analyses. Statistical testing within the chemometric analysis using the ChemRICH platform was determined by Kolmogorv-Smirnov testing and an FDR adjusted p-value (q-value) of less than 0.05 was considered significant. Individual metabolites achieving a q-value less than 0.05 were selected for individual analysis for their relationship with disease activity and clinical response to MTX. The Wilcoxon rank-sum test was used to compare changes in metabolites levels over the treatment period between patient groups based on clinical response based on ACR Pedi 70 or JADAS-71. Spearman’s correlations were used to evaluate the relationships between metabolite levels and disease activity by JADAS-71 and active joint counts. A multivariate nominal regression model analysis was used to explore multivariate associations with MTX response. Analyses were performed using JMP software version 11 (SAS Institute, Cary, NC).

## Results

### Patient Demographic and Clinical Data

Subjects were identified for inclusion in this analysis from an ongoing prospective cohort study on biomarkers of MTX efficacy in JIA. Subject selection was based on the availability of plasma samples both at baseline and after 3 months of MTX therapy at a stable dose of 15 mg/m^2^, as well as the availability of clinical response data defined using the ACR Pedi response criteria. To optimize discrimination, 2 groups comprised of extreme response phenotypes were selected. Specifically, 15 subjects were selected based on the achievement of ACR Pedi 70 response criteria by 3 months and were classified as “responders”, and 15 subjects were selected based on their failure to achieve ACR Pedi 30 response criteria by 3 months and were classified as “non-responders”. The demographics of each of the two groups are provided for comparison (Table 1). The cohort consisted of 30 subjects with a median age of 9.9 years (range: 1.8–17 years), with 21 females, 25 subjects on concomitant NSAID therapy, 2 receiving corticosteroids, and 18 receiving MTX *via* subcutaneous injection. No significant differences in baseline demographic characteristics nor baseline measures of disease activity were found between the groups. Of note, there were also no significant differences in baseline clinical characteristics with the remaining portion of the cohort excluded from this work (*n* = 38) including JIA subtype, age, gender, MTX route of administration, MD-VAS, PT-VAS, CHAQ, baseline active joint count, baseline JADAS-71, ESR or CRP. As expected, multiple measures of disease activity were significantly lower at the 3-months visit in the responder group compared to the non-responders, including: JADAS-71, active joint count, PT-VAS, MD-VAS, and CHAQ.

**TABLE 1:** Patient demographic and clinical data. Following 3-months of MTX therapy, patients meeting the ACR Pedi 70 response criteria (ACR Pedi >70) were considered responsive to MTX and patients failing to meet ACR Pedi 30 response criteria were considered non-responsive to MTX. The demographics, concomitant medications, route of MTX administration, and baseline and 3-months measures of disease activity are included and compared between the two groups (comparisons resulting in a *p-value* < 0.05 are italicized). Baseline clinical data are representative of the larger cohort from which these samples were chosen (data not shown).

	ACR pedi < 30	ACR pedi > 70	p-value
Patients, no	15	15	NA
Female, no. (%)	10 (67)	11 (73)	1.0
Age, years	10 [6,15]	9 [5,13]	0.48
NSAID, no. (%)	14 (93)	11 (73)	0.33
Corticosteroids, no. (%)	2 (13.3)	0 (0)	0.48
MTX, subcutaneous, no. (%)	7 (47)	11 (73)	0.26
***Baseline***	**ACR Pedi < 30**	**ACR Pedi > 70**	**p-value**
Active joints, no	6 [2,11]	6 [2,13]	1.0
PT-VAS	6 [3,8]	6 [3,8]	0.80
MD-VAS	4 [3,7]	5 [3,6]	0.97
CHAQ	0.4 [0.1.6]	0.7 [0.3.1.2]	0.36
ESR, mm/hr	10 [9,23]	16 [10,57]	0.10
CRP	0.6 [0.5.1.5]	1.5 [0.5.3.2]	0.10
JADAS-71	16 [13.24.6]	18.7 [10.6.34]	0.85
***3-months***	**ACR Pedi < 30**	**ACR Pedi > 70**	**p-value**
Active joints, no	5 [1,9]	0 [0,5]	*0.02*
PT-VAS	5 [3,7]	0 [0,2]	*<0.0001*
MD-VAS	3.5 [1.8.5]	0 [0,1]	*<0.0001*
CHAQ	0.4 [0.1.1]	0 [0,0]	*0.003*
ESR, mm/hr	9 [7.8.17.8]	9 [7,14]	0.76
CRP	0.5 [0.5.1.1]	0.5 [0.5.1.2]	0.31
JADAS-71	14.2 [7.8.17]	2 [0,7]	*0.0002*

**FIGURE 1:**
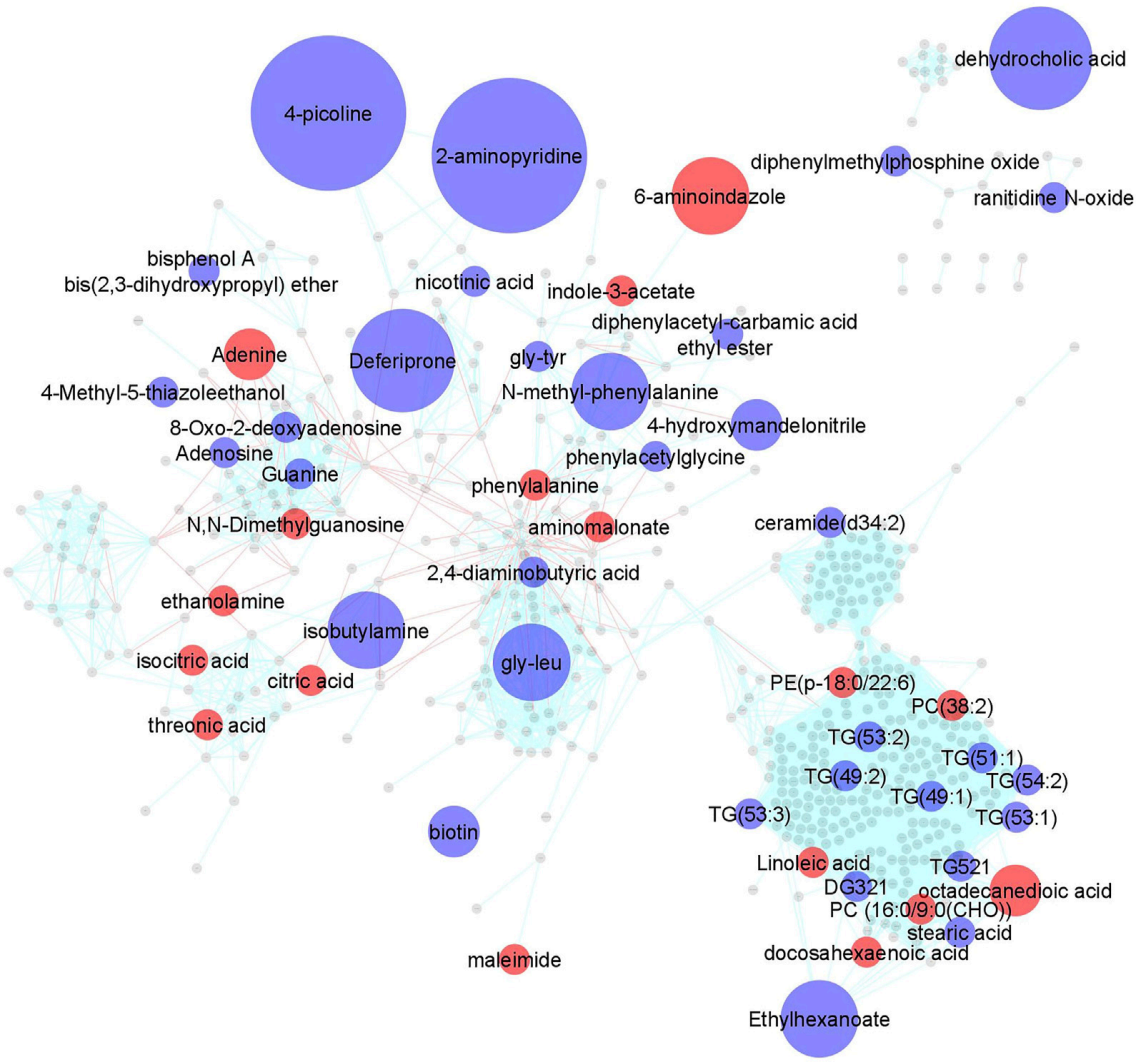
Changes in the plasma metabolomic profile in children with JIA following the initiation of MTX. Metabolite levels were determined at baseline and after 3 months of MTX therapy. Changes in metabolite levels were determined by paired analysis and metabolic changes were mapped using MetaMapp and visualized in Cytoscape 3.7.1. Metabolites found to significantly change based on a p-value < 0.05 were color coded and indicate either an increase (red) or decrease (blue) following initiation of MTX. Node size is proportional to measured fold-change. Red lines between nodes represent KEGG reaction pairs, while blue-green lines represent pairing based on chemical similarity.

Impact of MTX on the plasma metabolome in JIA. Using a semi-targeted global metabolomics approach, 673 identified metabolites were measured in plasma samples collected for each patient at baseline and at their 3-months visit. A paired analysis was conducted to evaluate metabolic changes over the treatment period and a metabolic network map was created using MetaMapp and visualized in Cytoscape 3.7.1 (Figure 1). As highlighted, 50 metabolites across a diversity of biochemical classes were impacted by MTX (*p* < 0.05). Further chemometric enrichment analysis by chemical class clustering was conducted using the ChemRICH platform and identified a class effect of MTX on unsaturated triglycerides. MTX treatment was found to be associated with a reduction in plasma levels of unsaturated triglycerides (q = 0.0009).

### Identification of Plasma Metabolic Markers Responsive to MTX Therapy in JIA

**FIGURE 2:**
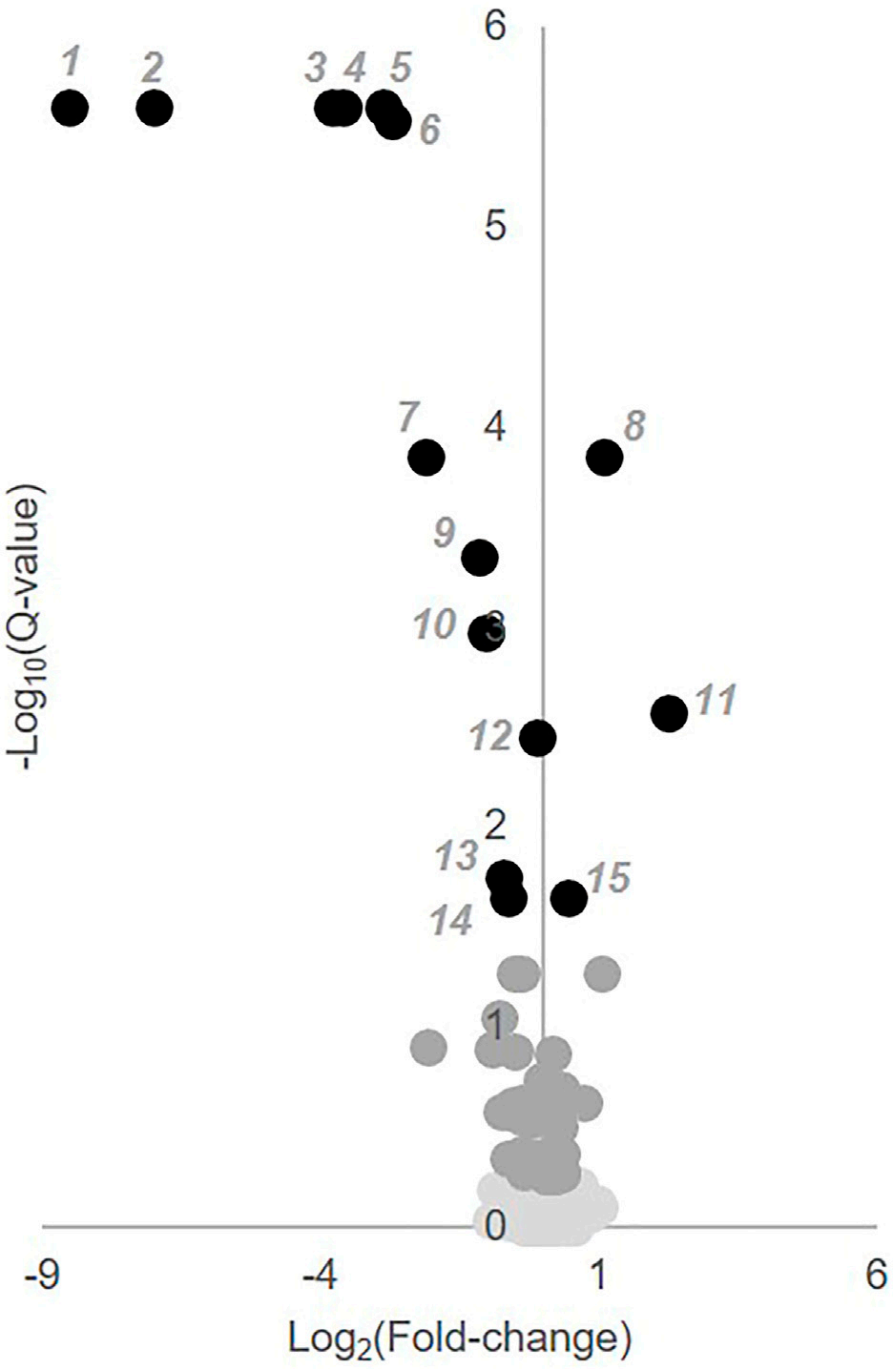
Identification of key metabolites altered following the initiation of MTX. Semi-quantitative data from 673 identified metabolites measured at baseline and after 3 months of MTX therapy were compared by paired univariate analysis. Metabolomics data was analyzed using MetaboAnalyst 3.0 and the resulting volcano plot of key metabolites based on a q-value < 0.05 are presented. In the volcano plot, black numbered metabolites represent those found to be altered following the initiation of MTX.

To identify individual metabolic markers that are responsive to MTX therapy, metabolites found to change over the treatment period with a q-value less than 0.05 were selected for further analysis (Figure 2). A total of 15 metabolites met these criteria and are listed along with their chemical class, fold-change, direction of change, coefficient of variation for both baseline and 3-months measurements, and q-value (Table 2). Plots for the semi-quantitative measurement of each of the identified metabolites based on log normalized peak intensity is provided for visual comparison (Figure 3).

**TABLE 2:** Plasma metabolites altered following 3-months of MTX therapy. The table of metabolites are identified by number from the volcano plot in Figure 2. The metabolite name, chemical class, fold-change color coded based on whether the metabolite increased (red) or decrease (blue), the coefficient of variation (CV) for the metabolite at the baseline and 3-months timepoint, and the q-value are provided.

#	Name	Class	Fold-change	Baseline CV	3-months CV	q-value
1	2-Aminopyridine	Pyridine	370	0.38	2.82	2.5 × 10^−6^
2	4-Picoline	Picoline	125	0.26	2.65	2.5 × 10^−6^
3	Dehydrocholic Acid	Bile Acid	14	0.39	1.83	2.5 × 10^−6^
4	Deferiprone	Pyridine	12	1.74	1.51	2.5 × 10^−6^
5	Isobutylamine	Butylamine	7.1	0.43	1.11	2.5 × 10^−6^
6	Ethylhexanoate	Fatty Acid	6.3	4.45	2.67	3.0 × 10^−6^
7	Gly-Leu	Dipeptide	4.3	0.70	1.02	1.4 × 10^−4^
8	Adenine	Purine	2.1	0.50	0.39	1.4 × 10^−4^
9	Biotin	Imidazole	2.2	0.34	0.55	4.5 × 10^−4^
10	4-Hydroxymandelonitrile	Acetonitrile	2.0	0.40	0.64	0.0011
11	6-Aminoimidazole	Indazole	4.8	1.45	0.67	0.0027
12	Ceramide (d34:2)	Ceramide	1.1	0.29	0.21	0.0036
13	Nicotinic Acid	Pyridine	1.6	1.79	3.88	0.018
14	TG (53:2)	Triglyceride	1.5	0.51	0.75	0.023
15	Maleimide	Maleimide	1.40	0.33	0.33	0.023

**FIGURE 3:**
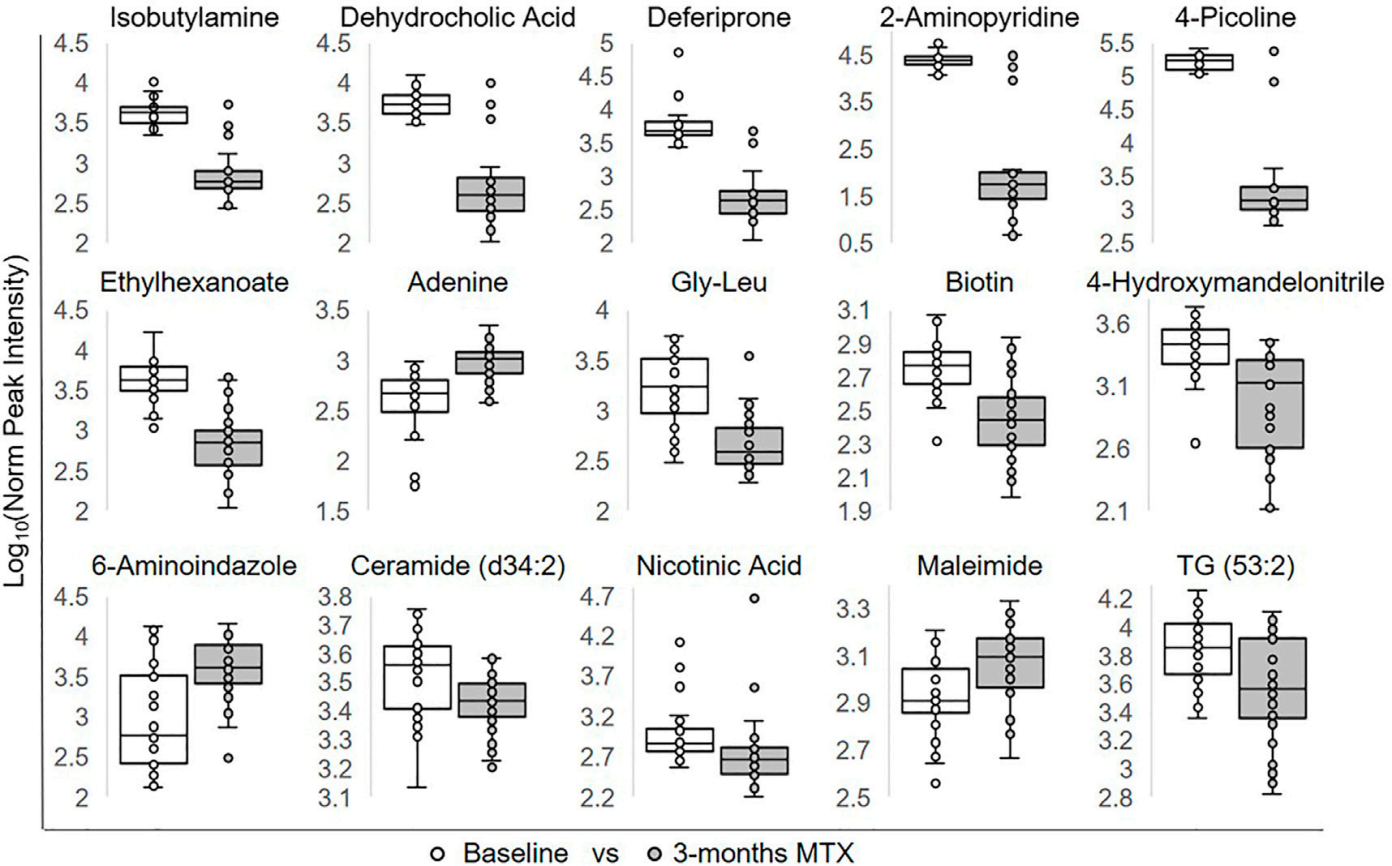
Relative plasma levels of metabolites significantly altered following the initiation of MTX. The log normalized peak ion intensity plots for the eight metabolites identified by univariate analyses are presented. Data points and representative box and whisker plots are shown based on metabolomics analysis at baseline and following the 3-months MTX treatment period. Log normalized peak ion intensities were compared between groups using paired non-parametric analysis with unequal variances.

### Association of Metabolic Markers With Disease Activity in JIA

**FIGURE 4:**
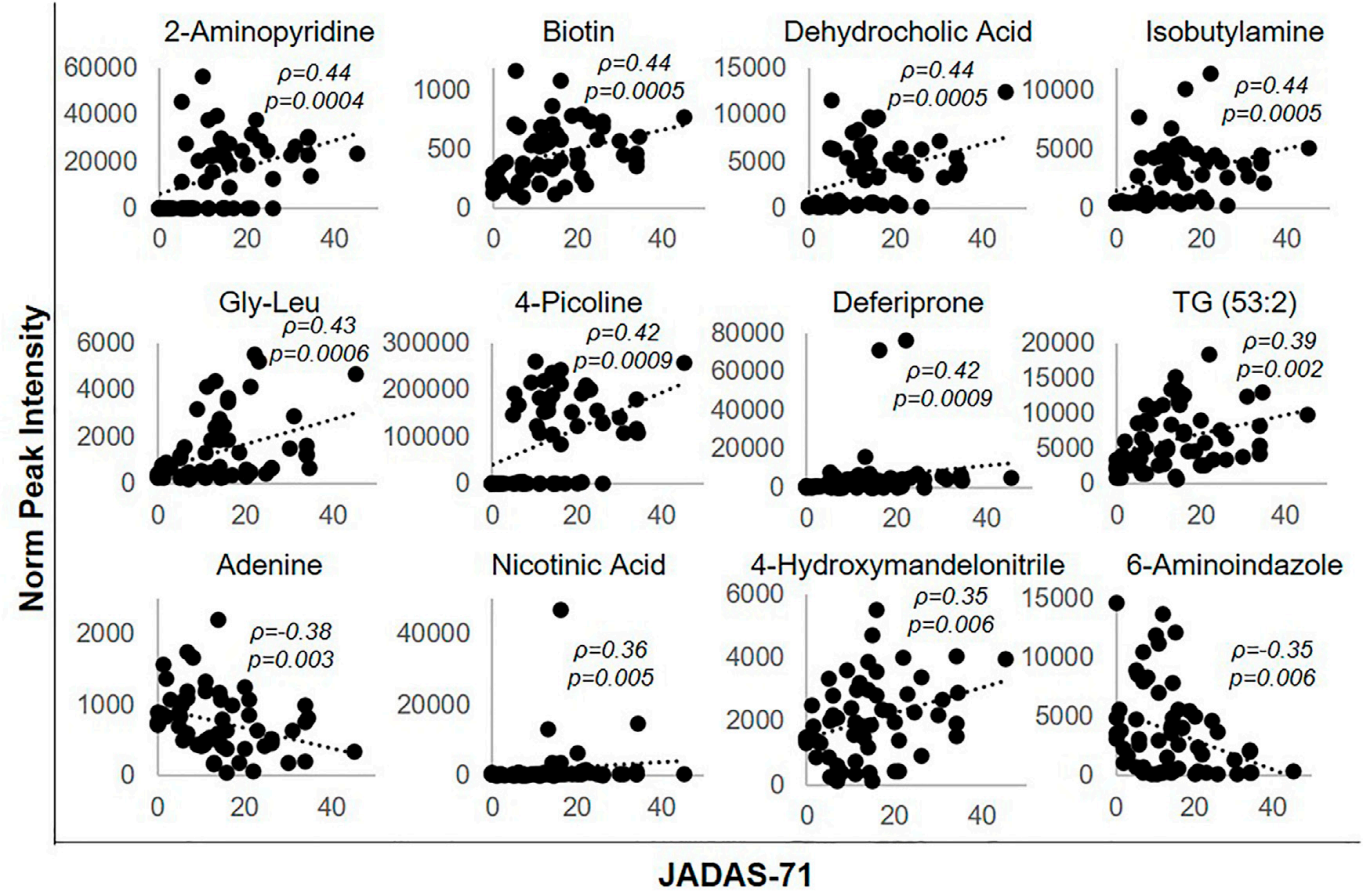
Association of disease activity with metabolite levels over the 3-months treatment period. The normalized peak ion intensity for each of the identified metabolites was evaluated for its relationship with JADAS-71 scores by Spearman’s rank correlation analysis. The resulting Spearman’s correlation coefficient (ρ) and associated p-values are provided.

Metabolites significantly impacted following the initiation of MTX were then evaluated for their association with disease activity using metabolite levels and JADAS-71 scores combined from both the baseline and 3-months visit (Figure 4). Lower disease activity scores were found to be significantly associated with lower levels of 2-aminopyridine (q = 0.002), biotin (q = 0.002), dehydrocholic acid (q = 0.002), isobutylamine (q = 0.002), glycine-leucine (gly-leu) (q = 0.002), 4-picoline (q = 0.002), deferiprone (0.002), triacylglycerol 53:2 (TG 53:2) (q = 0.004), nicotinic acid (q = 0.007), 4-hydroxymandelonitrile (q = 0.007), and higher levels of adenine (q = 0.005) and 6-aminoindazole (q = 0.007). For comparison, the correlation of the identified metabolites with JADAS-71 were stronger than those observed for serum CRP levels (*ρ* = 0.31, *p* = 0.31) and similar to those seen with the ESR (*ρ* = 0.45, *p* = 0.0004), which is a component of the JADAS-71 composite score calculation.

Similarly, the total number of active joint counts at baseline and 3-months were evaluated for their association with the identified metabolic markers. Lower active joint counts were associated with lower levels of 2-aminopyridine (*ρ* = 0.27, *p* = 0.03, q = 0.096), biotin (*ρ* = 0.33, *p* = 0.01, q = 0.06), dehydrocholic acid (*ρ* = 0.34, *p* = 0.007, q = 0.06), gly-leu (*ρ* = 0.26, *p* = 0.05, q = 0.096), and higher levels of adenine (*ρ* = -0.26, *p* = 0.05, q = 0.096) and 6-aminoindazole (*ρ* = -0.35, *p* = 0.006, q = 0.06). In contrast, active joint counts were not found to be significantly correlated with either serum CRP levels (*ρ* = 0.11, *p* = 0.41), or the ESR (*ρ* = 0.18, *p* = 0.17).

### Metabolic Markers Associated With MTX Efficacy in JIA

**FIGURE 5:**
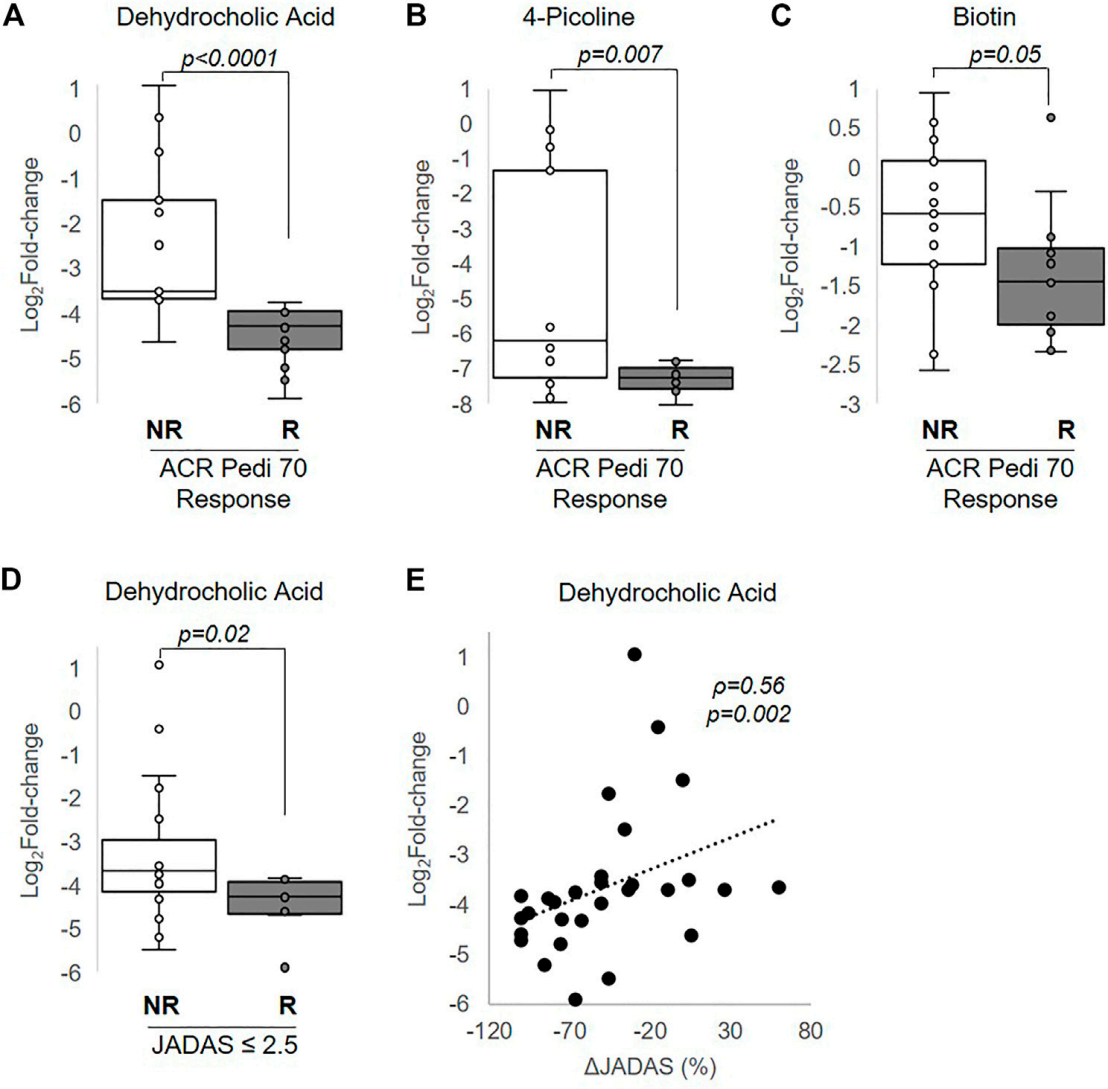
Association of changes in metabolite levels with MTX efficacy at 3 months. The fold-change in **(A)** dehydrocholic acid, **(B)** 4-picoline, and **(C)** biotin are compared between responders and non-responders based on achievement of ACR Pedi 70 response criteria. **(D)** The fold-change in dehydrocholic acid over the 3-months treatment period is compared between responders and non-responders based on achievement of a JADAS ≤2.5. **(E)** The association between fold-change in dehydrocholic acid and percentage change in JADAS-71 was evaluated by Spearman’s rank correlation analysis. The resulting Spearman’s correlation coefficient (ρ) and associated p-values are provided. Unpaired analysis in **(A–D)** were conducted by Wilcoxon rank-sum analysis and the resulting p-values are provided.

MTX efficacy in this study was defined as achieving a 70% improvement in disease activity based on the ACR Pedi 70 response criteria by 3 months. Non-responders were defined as those not even achieving ACR Pedi 30 response criteria. Changes in metabolite concentrations over 3 months following the initiation of MTX were compared between patients classified as responders and non-responders. Of the metabolites found to be significantly altered following the initiation of MTX, only changes in three of these metabolites were found to be associated with MTX response and included: dehydrocholic acid (q = 0.0003), 4-picoline (q = 0.05), and biotin (q = 0.24) (Figure 5). Low disease activity at 3 months, defined as a JADAS-71 score of ≤2.5, was used as a secondary measure of MTX response. Of the metabolites found to be significantly associated with ACR Pedi 70 response at 3 months, only changes in dehydrocholic acid were found to be significantly associated with achieving a JADAS-71 ≤ 2.5 (q = 0.06) (Figure 5D). Further, percent change from baseline in JADAS-71 was found to be positively associated with the observed reduction in dehydrocholic acid (Figure 5E).

Although there was no statistically significant association of route of administration with clinical response (Table 1), MTX route of administration has previously been demonstrated to impact clinical response to MTX (Visser and Van der Heijde, 2009). As a result, the JIA population was further stratified by dosing route to investigate if the observed metabolic changes associated with response varied by route of administration. Among patients receiving MTX orally, ACR Pedi 70 response at 3 months continued to be associated with greater reductions in dehydrocholic acid (*p* = 0.009), 4-picoline (*p* = 0.04), and biotin (*p* = 0.04). However, among patients receiving MTX by the subcutaneous route, ACR Pedi 70 response at 3 months was only associated with greater reductions in dehydrocholic acid (*p* = 0.009). A nominal logistic regression model for MTX response by ACR Pedi 70 criteria was built using route of administration and change in dehydrocholic acid levels as the independent variables to evaluate if the relationship of dehydrocholic acid levels with clinical response were independent of differences in response based on route of administration. MTX route of administration was not found to be significantly associated with response, but greater reductions in dehydrocholic acid continued to demonstrate a significant association with response (*p* = 0.03) in the model.

## Discussion

In this work, the initiation of MTX in children with JIA is found to be associated with a diversity of changes in the plasma metabolome. Visualization by metabolic network mapping highlights these changes (Figure 1). Alterations in the plasma metabolome reflect direct effects of MTX on these metabolic pathways, or alternatively may reflect alterations in the immunoinflammatory disease process. Metabolic pathways predicted to be impacted based on the known pharmacological activity of MTX include pathways related to folate metabolism, including intermediates of nucleotide metabolism and methylation (Cronstein and Aune, 2020). In our study, treatment with MTX was found to be associated with altered nucleotide metabolism, including increased plasma adenine and N,N-dimethylguanosine levels, and decreased levels of adenosine, guanine, and 8-oxo-2-deoxyadenosine. In contrast to previous work that found no difference in adenosine concentrations in the blood of children with JIA receiving MTX (Dolezalova et al., 2005), this work found a 40% reduction in plasma adenosine levels following the initiation of MTX. This finding is contradictory to those expected based on the hypothesis that MTX mediates its anti-inflammatory activity through the indirect inhibition of adenosine deaminase resulting in an increase in extracellular adenosine concentrations (Nalesnik et al., 2011). However, it must be noted that adenosine is unstable in plasma and these samples were not prepared in such a manner to control for this rapid degradation (Lofgren et al., 2018). MTX treatment was also associated with an increase in the amino acid phenylalanine with a corresponding reduction in its N-methylated product, which represents a potential indicator of reduced methyltransferase activity. However, no significant reduction in plasma levels of the universal methyl donor S-adenosyl-methionine were observed (Wang and Chiang, 2012).

The majority of metabolites identified as significantly altered following the initiation of MTX haven’t been previously identified as pharmacological targets of MTX. It is possible that changes in many of these metabolites are reflective of metabolic changes related to the underlying immunoinflammatory process. The chemical enrichment analysis found that the only class-wide metabolic effect associated with MTX treatment was a reduction in unsaturated triglycerides. This finding is in agreement with previous studies demonstrating that children with active JIA had, on average, 37–59% higher plasma triglyceride concentrations compared to healthy control subjects, and that inactive disease was associated with normalization of these levels (Ilowite et al., 1989; Tselepis et al., 1999). Elevated triglycerides represent a component of a broader dyslipoproteinemia of autoimmunity attributed to reduced lipase activity related to pro-inflammatory signaling and associated with increased atherogenic risk (Wang et al., 2020). Our work also found MTX therapy was associated with increases in plasma concentrations of several unsaturated fatty acids, including docosahexanoic acid and linoleic acid. Previous work has similarly found lower unsaturated fatty acid levels in the serum of JIA patients with active disease, and found that unsaturated fatty acid concentrations were inversely associated with markers of disease activity (Gorczyca et al., 2017). The omega-3 unsaturated fatty acids have been proposed to alter eicosanoid metabolism in favor of an anti-inflammatory state and potentially alter innate and adaptive immune cell function, as well as cytokine and reactive oxygen species production (Miles and Calder, 2012) (Gheita et al., 2012).

In evaluating potential metabolic biomarkers associated with MTX response, only metabolites achieving an FDR-adjusted p-value (i.e., q-value) less than 0.05 were considered. The resulting metabolites represented a diversity of biochemical classes, with many representing metabolites that fail to map to endogenous metabolic pathways. Several of these metabolites were found to be significantly associated with JADAS-71 scores and active joint counts resulting in associations that were stronger than those observed with ESR and CRP, which represent the inflammatory markers commonly used as measures of active disease in practice. In comparing changes in metabolite levels based on the ACR Pedi response criteria, changes in dehydrocholic acid, 4-picoline, and biotin were found to be significantly different between responders and non-responders; and in subjects who achieved a JADAS ≤2.5, changes in dehydrocholic acid were found to be significantly different. Furthermore, changes in dehydrocholic acid were found to be significantly associated with changes in JADAS over the treatment period and remained significant after controlling for MTX administration. None of these metabolites have been previously associated with the efficacy of MTX and therefore represent potential novel biomarkers of response.

Dehydrocholic acid is a secondary bile acid and is an oxidation product of cholic acid (Navarro Suarez et al., 2018). Recognizing that secondary bile acids are primarily formed through enterohepatic recirculation of primary bile acids with metabolism occurring *via* the gut microbiota (Staley et al., 2017), changes in dehydrocholic acid levels secondary to MTX therapy support the findings that MTX efficacy in autoimmune arthritis may be related to its effect on the gut microbiota (Artacho et al., 2020; Scher et al., 2020; Nayak et al., 2021; Wu, 2021). This potential relationship is further supported by the identification of biotin as a metabolite associated with MTX efficacy, as systemic biotin is at least partially generated through gut microbial metabolism (Rowland et al., 2018). 4-picoline is within the class of compounds known as methylpyridines and is an aromatic compound found in food stuffs and its presence may represent consumption of foods containing this compound (Arn and Acree, 1998). The basis for the relationship between MTX efficacy and 4-picoline levels will require further investigation, as its role in metabolism is unknown and has only been speculated to represent a potential biomarker of foods enriched in this metabolite, such as tea.

This is the first study to evaluate the relationship between the effect of MTX on the plasma metabolome in relation to its clinical effect. Previous metabolic studies have been conducted in RA and have identified a number of metabolites and metabolic pathways associated with clinical response to MTX, however these studies were largely based on metabolomic analysis at a single timepoint. An NMR-based metabolomics analysis of serum samples from 38 RA patients found significant differences in the concentrations of 11 metabolites between MTX responders and non-responders when measured after 24 weeks of therapy (Wang et al., 2012). This list included markers of nucleotide (uric acid, uracil, hypoxanthine), amino acid (aspartate, methionine, histidine, glycine, tryptophan, taurine), TCA cycle (α-oxoglutarate) and microbial (TMAO) metabolism. The same metabolic pathways were also found to be impacted by MTX in our study, however the metabolites representing these changes differed in our pediatric cohort. This may reflect differences in the study design, in that the previous study only evaluated metabolite concentrations at the 24-weeks timepoint, while our study focused only on metabolites that changed in response to MTX therapy over a 12-week period. Similar efforts have focused on using baseline metabolomic profiling to identify predictors of drug response (Gosselt et al., 2020). Responders were found to have significantly lower concentrations of several markers of nucleotide and amino acid metabolism (guanosine diphosphate, adenosine triphosphate, uric acid, taurine, homocysteine) and higher concentrations of intermediates of glycolysis (glycerol-3-phosphate, diphosphoglycerate, phosphoenolpyruvate). These findings suggested that pretreatment variability in amino acid metabolism and cellular respiration may predict future response to MTX therapy, but the work did not explore how MTX impacted the metabolome and whether the impact on the metabolome was associated with its efficacy. In fact, the authors highlighted the need for future longitudinal metabolomic studies to investigate the impact of MTX on metabolism in relation to efficacy with a goal of improving our understanding of the mechanism of action of MTX, as done here. Another study focused on an untargeted approach that measured over 3,000 lipid metabolites at baseline and after 4 weeks of therapy to predict MTX response at 6 months in RA (Maciejewski et al., 2021). The study failed to identify any lipid species, alone or in combination, that predicted response to MTX. Again, this study focused on predictive markers of MTX response and used an untargeted approach limited to lipid metabolites. Ultimately, our study confirms that MTX has a significant impact on the plasma metabolome in children with JIA, and that some of these changes differentiate patients responding to MTX from those not responding to MTX. Specifically, the metabolites identified here implicate exogenous and microbiota metabolites, and highlight the potential role of environmental and gastrointestinal microbial metabolism as important mediators in the clinical response to MTX.

The limitations of this study include an exploratory study design with a limited sample size of 30 JIA patients that was further divided into two groups based on clinical response. This sample size was in line with previous metabolomics studies and power was increased through the use of a paired approach to the analysis using both baseline and 3-months follow up samples from the same patients. Choosing extreme response phenotypes intended to clearly delineate the clinical response differences between groups and minimize the known heterogeneity in JIA. The baseline clinical features however did not differ from the larger cohort. In addition, the identification of statistically significant metabolomic changes and metabolic biomarkers demonstrates that the study was adequately powered for these analytes. Another limitation of the study is the variation in the route of MTX administration in the study cohort. This could result in potential confounding variables related to variation in MTX bioavailability, metabolomic changes, and clinical response. Although the subcutaneous route is preferred, especially for higher doses of MTX, such as the 15 mg/m^2^ dose used in this study, a national shortage of injectable MTX resulted in the use of oral MTX in 12 of our patients. Although we did not find a statistically significant difference in response based on route of administration, there was increased use of subcutaneous MTX in responders (73%) compared to non-responders (47%). We attempted to control for route of administration in our sub-analysis, which ultimately resulted in a reduction in power, but we were able to confirm the relationship of reductions in dehydrocholic with clinical response by both routes of administration. Similarly, although use of NSAIDs and corticosteroids did not statistically differ between responders and non-responders, it must be acknowledged that these therapeutic agents can also impact the plasma metabolome and may represent potential confounding factors in this analysis. Only 2 subjects received corticosteroids in this cohort, but were not actively on corticosteroids at the time of baseline or 3-months sampling (i.e., they received and were discontinued on corticosteroids within the 3-month sampling window). In addition, recognizing that several of the metabolites that we found were associated with the gut microbiota, it may have been helpful to collect information on antibiotic use prior to initiation of the study and any prospective dietary changes throughout the study. The effect of diet and diurnal variation on the plasma metabolome may represent additional confounding variables, as samples were collected as part of routine care and were not controlled for either diet or time of day. However, recent work investigating the impact of season, time of day, fasting status, tobacco, alcohol, and NSAID use failed to demonstrate a significant impact of these potential confounding variables on the metabolome, but did find that the metabolome was sensitive to sex and age (Hardikar et al., 2020). Although we were not able to capture all potentially confounding variables in our analysis, our responder and non-responder groups were found to be similar in both sex and age. The study is also limited by the semi-quantitative approach to measuring metabolite levels that lack authentic internal standards for the absolute quantitation of each of the identified metabolites. However, this is the standard approach in global metabolomics analyses. Although multiple analytical platforms were used to capture a diverse representation of the plasma metabolome, there remains a multitude of unidentified metabolites that were not included in this analysis. However, this limitation applies to all metabolomics studies.

## Conclusion

Metabolomics represents an unbiased and untargeted approach to improve our understanding of the underlying biochemical changes associated with MTX therapy and the relationship of these changes with its clinical efficacy in JIA. As a result, metabolomics serves as a tool for hypothesis generation that holds the possibility of both identifying novel drug target pathways for the treatment of JIA as well as the identification of potential metabolic biomarkers to guide clinicians in the optimization of MTX therapy in JIA. The findings in this study support a robust and diverse change in the plasma metabolome following the initiation of MTX in children with JIA. In particular, MTX therapy is associated with a class-wide reduction in plasma triglycerides that is likely reflective of the previously described dyslipoproteinemia of autoimmunity that is at least partially corrected following the initiation of MTX. Although a number of individual metabolites representing a diversity of chemical classes were found to be significantly associated with disease activity, only a few metabolites were found to discriminate between patients based on clinical response to MTX. These metabolites included dehydrocholic acid, biotin, and 4-picoline, and are all likely related to exogenous sources and possibly related to gut microbial metabolism. Subsequent targeted and functional metabolic studies are needed to further evaluate these putative biomarkers of MTX response in JIA.

## Data Availability

The datasets presented in this study can be found in online repositories. The names of the repository/repositories and accession number(s) can be found at: https://www.metabolomicsworkbench.org/ under Project ID: PR001148.
